# Walker Parameter for Mean Stress Correction in Fatigue Testing of Al-7%Si-Mg Alloy Castings

**DOI:** 10.3390/ma10121401

**Published:** 2017-12-08

**Authors:** Hüseyin Özdeş, Murat Tiryakioğlu

**Affiliations:** School of Engineering, University of North Florida, 1 UNF Drive, Jacksonville, FL 32224, USA; huseyinozdes@gmail.com

**Keywords:** mean stress effect, runout analysis, S–N curve, Basquin Law

## Abstract

In this study, performance of two existing Walker parameter estimation models has been investigated. Results show that those developed mainly for steel did not provide reasonable fits to experimental Walker parameters for fatigue data for Al-7%Si-Mg alloy castings in the literature. A strong relationship between the Walker parameter and the structural quality, quantified by the quality index, Q_T_, was observed and an empirical equation to estimate the Walker parameter for these alloys was developed. These findings indicate that the Walker parameter is not an intrinsic material property and the structural quality of the specimens must be taken into account for mean stress correction in fatigue testing.

## 1. Introduction

High cycle fatigue (HCF) performance of metals has been studied extensively in the literature. The change in fatigue life, N_f_, with stress amplitude, σ_a_, is known to follow the Basquin law [1]:

(1) $$\sigma_{a}{=\sigma }_{f}^{\prime }N_{f}^{b}$$

where, $\sigma_{f}^{\prime }$ is fatigue strength coefficient and b is the Basquin exponent. The Basquin law has been used to express the high cycle fatigue (HCF) behavior of metals. Both Basquin parameters are strongly affected by the material [2], specimen geometry [3] as well as the type of test conducted [4]. It is well-known that mean stress has a significant effect on the HCF behavior [5]. When the mean stress is compressive, fatigue life is longest for the same level of alternating stress. When mean stress is tensile, fatigue life is lowest. For zero mean stress, fatigue life is between the two other S–N curves. In the literature, numerous correction methods have been introduced to account for the mean stress effect on fatigue test results. These models have been developed to modify the alternating stress and find the equivalent stress amplitude, σ_eq_, at zero mean stress. Among those, one of the most widely used models is the one developed by Walker [6], in which the equivalent stress amplitude is found as:

(2) $$\sigma_{\mathrm{eq}}{=\sigma }_{a}{\left(\frac{2}{1-R}\right)}^{\gamma }$$

where R is the load ratio, (minimum to maximum stress ratio) and γ is the Walker parameter, which is an empirical parameter that changes between 0 and 1, representing the maximum and minimum sensitivity to the load ratio, respectively [7]. At zero mean stress, R = −1, and consequently, σ_eq_ = σ_a_. The Walker equation was reported [8,9] to provide best fits in metals. There have been several studies [6,9,10,11] in which the Walker parameter was treated as a material property. To the authors’ knowledge, there has not been any effort to determine whether γ is affected by the structural quality of components. This study is motivated to fill this gap.

## 2. Background

In aluminum castings, structural defects, such as pores and oxide films entrained from surface, i.e., bifilms, have a strong, deleterious effect on the mechanical properties related to fracture, such as elongation [12], fracture stress [13,14], fracture toughness and fatigue life [15,16]. Davidson et al. [17] showed that there is a linear relationship between logarithms of fatigue life and largest defect size in Al-7%Si-Mg alloy castings. Moreover, one of the authors [18,19] demonstrated that the fatigue life distribution can be directly linked to the size distribution of largest defects in Al-7%Si-Mg alloy castings.

Iben Houria et al. [20] investigated the combined effect of largest defect size and mean stress on the fatigue strength (at 10^7^ cycles) of A356 alloy castings. At zero mean stress (R = −1), fatigue strength was not affected by the largest pore sizes less than 400 μm. With increasing largest pore size after 400 μm, however, there was a sharp decrease in fatigue strength. When only tensile stresses are present (R ≥ 0), the sensitivity of fatigue strength to maximum pore size was found to start at almost 50 μm. At pores sizes, larger than 400 μm, fatigue strength was not affected by maximum pore size. Hence the response of the aluminum casting with defects to alternating loads is completely opposite of each other at the two different stress ratios. This difference may be explained by the effect of the driving force of local stress concentrations at the tip of the crack during crack propagation, as observed by Gall et al. [21], who conducted FEM analysis of fatigue behavior in the presence of pores and hard inclusions embedded in a metal matrix. At R = −1 (fully-reversed), the contributions of pores and hard inclusions to driving force for crack propagation were identical. The contribution of inclusions was twice of that of pores when mean stress became positive. Therefore, there is evidence in the literature that fatigue behavior in the presence of structural defects, such as pores, is determined by mean stress. Consequently, the assumption made in correction models for mean stress that the contribution of structural defects is the same regardless of R, is not necessarily valid for parts with different structural defects and varying structural quality, such as castings.

Tensile testing is the most commonly used test to determine the mechanical properties of metals, such as yield strength (σ_Y_), tensile strength (S_T_) and elongation (e_F_). Tiryakioğlu and coworkers collected hundreds of data points from the aerospace and premium castings literature for Al-7Si-Mg, A206 and A201 alloy castings [22,23,24] and plotted elongation versus yield strength, which is minimally affected by structural defects. They concluded that the highest elongation points form a linear trend, which can be written as:

(3) $$e_{F(\max)}{=\beta }_{0}-\beta_{1}\sigma_{Y}$$

where β_0_ and β_1_ are alloy-dependent coefficients. For cast Al-Si-Mg alloys, β_0_ and β_1_ are 36.0 and 0.064 (MPa^−1^), respectively. Equation (3) can be used to estimate the ductility potential of aluminum and magnesium alloy castings [23,24,25,26,27]. The structural quality of aluminum alloy castings can then be quantified by using a quality index, Q_T_, which is expressed as:

(4) $$Q_{T}=\frac{e_{F}}{e_{F(\max)}}=\frac{e_{F}}{\beta_{0}-\beta_{1}\sigma_{Y}}$$

The authors have found [28,29] a strong relationship between Q_T_ and fatigue life in cast aluminum alloys, and demonstrated that Q_T_ can be used to predict fatigue strength [30,31] and Basquin coefficients for Al-Si-Mg-(Cu) alloy castings [31].

Turning our attention to the Walker parameter, Dowling et al. [9] showed that the Walker parameter, γ, changes linearly with tensile strength of steels:

(5) $${\gamma =\gamma }_{0}-{a\cdot S}_{T}$$

Dowling et al. reported γ_0_ and a to be 0.883 and 2 × 10^−4^ MPa^−1^, respectively, for steels. The same authors stated that γ = 0.651 when S_T_ is between 300 and 350 MPa, and γ = 0.473 for S_T_ values between 450 and 600 MPa. Lv et al. [8] stated that γ is expected to be low for materials with low ductility and proposed the following equation to determine the Walker parameter:

(6) $$\gamma =0.5\pm \frac{S_{T}-\sigma_{Y}}{S_{T}{+\sigma }_{Y}}$$

Lv et al. did not provide a physical reason for the ± sign but showed that there is agreement between the γ values reported by Dowling et al. [9] and those estimated by Equation (6).

In the studies summarized above, γ has been stated as a material property [6,9,10]. However, the results of Iben Houria et al. [20] and Gall et al. [21] on the fatigue behavior in aluminum castings with defects suggest that the effect of the mean stress around the structural defect that leads to fatigue failure is affected by the type of defect and the level of the mean stress. Consequently, it is hypothesized that the Walker parameter is not an intrinsic material property and is affected by the extrinsic effects of casting’s structure, i.e., casting quality. This hypothesis will be tested by using data from literature.

## 3. Analysis of Data from Literature

Data from seven studies [15,32,33,34,35,36,37] involving nine datasets for cast Al-7%Si-Mg alloys published in the literature with various R ratios have been reanalyzed. The datasets used in this analysis are as follows:

- Palmer [32] tested 356-T6 lost foam castings with as-cast and machined surfaces. Specimens with as-cast surfaces were tested at seven R ratios ranging from −1 to 0.44, whereas eight R ratios between −1 and 0.62 were used for machined specimens.
- Wang et al. [15] used Sr-modified A356 casting alloy in T6 heat treatment condition to study the influence of casting defects on the room temperature fatigue performance by using unnotched polished cylindrical specimens. R ratio of 0.1 and −1 have been included in the study.
- Koutiri et al. [33] studied the high-cycle fatigue behavior of cast hypo-eutectic Al-Si alloy to investigate the fatigue damage mechanisms under complex loading conditions with two different load ratios (R = 0.25 and 0.73).
- Oswalt [34] investigated unchilled test bars of 357-T6 alloy to determine the fatigue strength at R ratios of 0.2 and −1 were used in the study.
- Mu et al. [35] used AS7G06 (A357) cast aluminum alloy in T6 heat treatment condition for two different R ratios (0.1 and −1) to analyze types of defects at the origin of the failure.
- Munoz [36] focused on A357-T6 cast aluminum alloy to investigate the effect of the material microstructural parameters that affect the small fatigue cracks. R ratios of 0.1 and −1 were used in this study.
- Jana et al. [37] studied cast F357 (Be-free variant of A357) plates of ~3.3 mm thickness to investigate the effect of friction stir processing (FSP) on fatigue life of sand castings. Fatigue tests were run at stress ratio of R = 0 and R = −1 both before and after friction stir processing (FSP).

To quantify their structural quality levels, quality index values of each datasets were estimated by using equation 3 based on their tensile properties. In addition, the analysis conducted in this study incorporated a maximum likelihood (ML) method developed by Sarkani et al. [38] except for dataset from Palmer [32] who published their Basquin and Walker parameters. Though it was not mentioned above for the sake of clarity, it is known that varying stress ratios not only generate beneficial or detrimental effects on fatigue results, but also do act significantly different at HCF or LCF regions. Unsurprisingly, they have more noteworthy influence within the HCF region. In HCF region, damage is driven by elastic deformation meaning that fatigue life is mostly spent on crack initiation. Since there is not significant plastic deformation in this region, once crack initiated typically is followed closely by sudden fracture. Tensile mean stresses then could be observed more clearly in this region. Typically, due to time concerns in fatigue testing, tests are interrupted after certain cycles and the data points referred to as runouts. In some cases, these runouts are discarded from the analyses or treated as failure data which may cause misleading results. To this aspect of their significance, there has been some attempt made by researchers with statistical basis. To the authors’ knowledge, such a study with proper treatment of survival data had not been conducted before.

## 4. Results and Discussion

Tensile data, estimated Basquin parameters and the value of the Walker parameter are provided in Table 1. Note that the Basquin parameters were not estimated for 356-T6 castings by Palmer, who reported the estimated values of the Walker parameter for his datasets.

**Figure 1:**
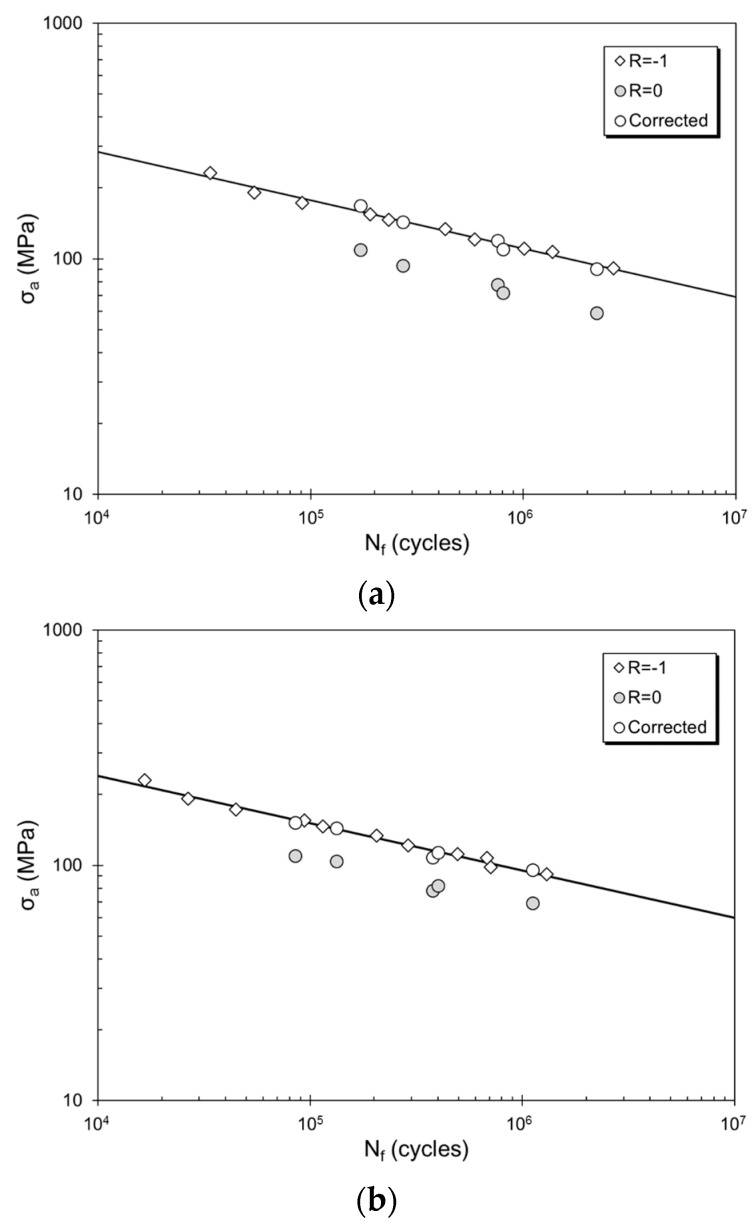

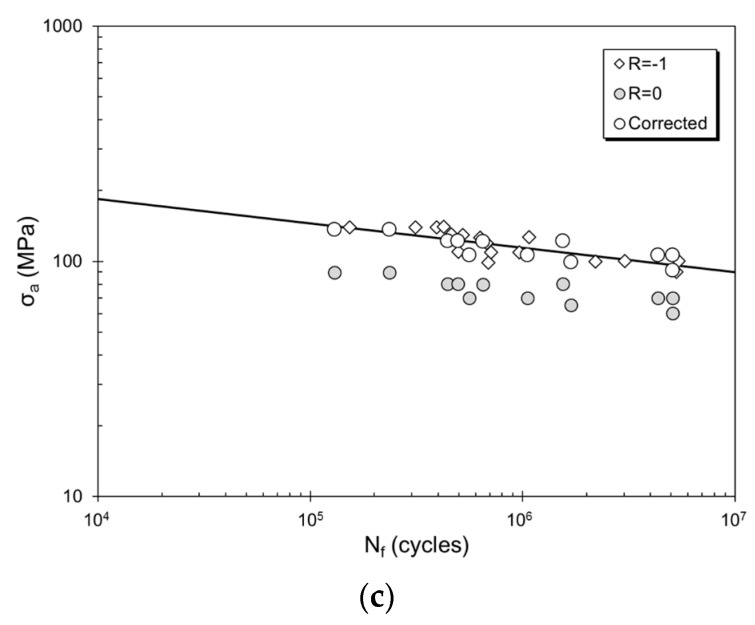
The S–N curves at R = −1 after Walker mean stress correction for (**a**) sand cast F357 by Jana et al. [37], (**b**) A357 by Mu et al. [35], and (**c**) friction stir processed F357 by Jana et al. [37]. The original data for R = 0 as well as their correction according to Walker equation (Equation (2)) are also indicated.

Note that Q_T_ values for each dataset were also provided, and ranged between 0.027 and 0.860, representing almost the entire spectrum (between 0 and 1) of the quality index. The S–N diagrams for three of the datasets before and after the Walker correction for mean stress are presented in Figure 1. These datasets are specifically chosen to represent the minimum, maximum and intermediate level of Q_T_ among the nine datasets investigated in this study. Regardless of the level of the structural quality level, the Walker correction provides very respectable fits to the experimental data.

**Figure 2:**
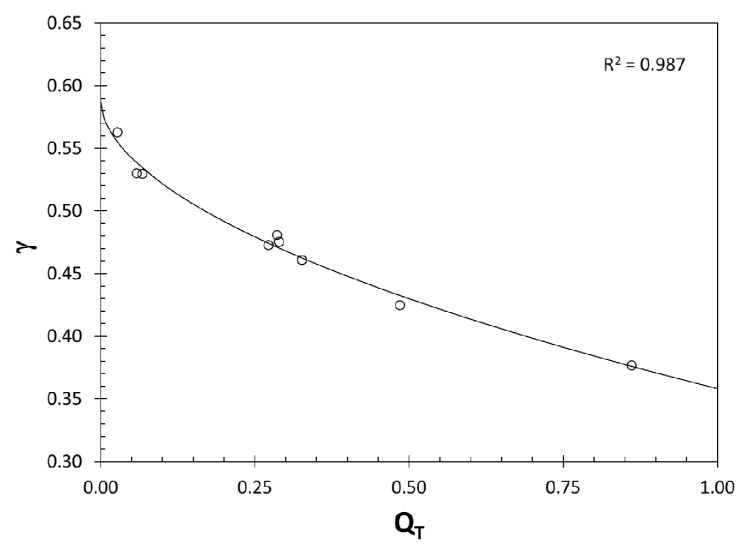
The change in γ as a function of Q_T_.

The hypothesis that the value of γ is affected by the structural quality of the castings has been tested; Q_T_ versus γ plots is presented in Figure 2.

Note that there is strong evidence that the Walker parameter is affected by the structural quality of aluminum castings. The best fit curve indicated in Figure 2 is expressed as;

(7) $$\gamma =0.588-0.230\cdot Q_{T}{}^{0.541}$$

with a coefficient of determination, R^2^ = 0.987. Hence with increasing quality, γ decreases and therefore castings become more sensitive to the mean stress effects in fatigue testing. Moreover, the highest quality Al-Si alloy casting (Q_T_ = 1.0) would have a Walker parameter of 0.358, which represents the intrinsic value for these alloys. As stated above, the Walker parameter has been regarded as a material property in the literature. However, the strong effect of Q_T_ on the Walker parameter found in this study indicates that γ includes the quality of the material and therefore has an extrinsic component.

**Figure 3:**
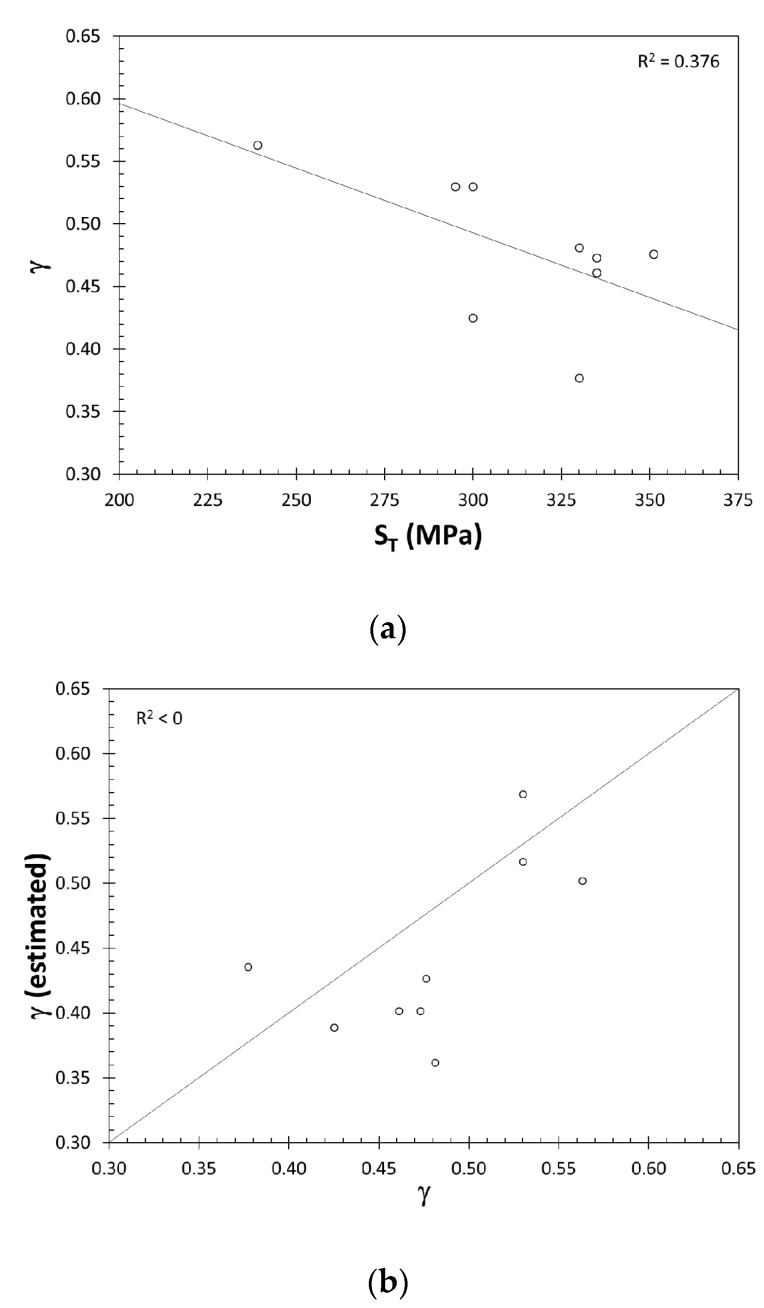
(**a**) The relationship between γ and S_T_, as suggested by Dowling et al. [9], and (**b**) the comparison of Walker parameters for best fit and estimated by using the equation proposed by Lv et al. [8].

The performance of the equations proposed by Dowling et al. [9] and Lv et al. [8] performed with the results summarized in Table 1 are presented in Figure 3.

The best fit line in Figure 3a is written as

(8) $$\gamma =0.803-1.0\times 10^{-3}S_{T}$$

However, the coefficient of determination is much lower and therefore the change in S_T_ can explain only 37.6% of the change in the Walker parameter. The equation proposed by Lv et al. was found to perform very poorly on cast Al-7%Si-Mg alloys, as shown in Figure 3b, as indicated by the R^2^ being lower than zero.

## 5. Conclusions

In the light of observations made in this study, following conclusions could be drawn:

- Experimental Walker parameter has been observed to show a correlation with Q_T_ (derived from tensile properties) meaning that it has an extrinsic component and should not be treated as a material property without considering the casting’s quality level.
- Equations, developed mainly for steel, to estimate the Walker parameter do not provide accurate results for Al-7%Si-Mg alloy castings.
- A new methodology for estimating the Walker parameter based on alloys quality level involving proper runout analysis has been developed. The following empirical equation can be used to estimate γ:
  $$\gamma =0.588-0.230\cdot Q_{T}{}^{0.541}$$

## Figures and Tables

**Table 1:** Tensile data, quality index and estimated Basquin and Walker parameters for the nine datasets.

Alloy	Ref.	σ_Y_ (MPa)	S_T_ (MPa)	e_F_ (%)	Q_T_	$\sigma_{f}^{\prime }$ (MPa)	b	γ	Notes
356	[32]	257	295	1.3	0.067			0.530	Machined
		238	239	0.6	0.027			0.563	As-cast
A356	[15]	240	300	10.0	0.485	904.8	−0.141	0.425	
A356 + 0.5Cu	[33]	250	330	5.7	0.285	1061	−0.180	0.481	
357	[34]	303	351	4.8	0.289	989	−0.213	0.476	Aerospace castings
A357	[35]	275	335	5.0	0.272	480	−0.104	0.473	
A357	[36]	275	335	6.0	0.326	768	−0.141	0.461	
F357	[37]	238	239	0.6	0.027	1529	−0.201	0.563	No FSP
		290	330	15.0	0.860	1877	−0.205	0.377	FSP
